# The efficacy of non-anesthesiologist-administered propofol sedation with a target-controlled infusion system during double-balloon endoscopic retrograde cholangiopancreatography

**DOI:** 10.1186/s12876-023-02936-8

**Published:** 2023-09-04

**Authors:** Kazuya Miyamoto, Kazuyuki Matsumoto, Taisuke Obata, Ryosuke Sato, Akihiro Matsumi, Kosaku Morimoto, Taiji Ogawa, Hiroyuki Terasawa, Yuki Fujii, Tatsuhiro Yamazaki, Daisuke Uchida, Shigeru Horiguchi, Koichiro Tsutsumi, Hironari Kato, Motoyuki Otsuka

**Affiliations:** Department of Gastroenterology and Hepatology, Okayama University Hospital, 2-5-1 Shikata-Cho, Okayama, 700-8558 Japan

**Keywords:** Balloon-assisted endoscopy, Propofol, Diazepam, Sedation

## Abstract

**Background:**

The sedation method used during double-balloon endoscopic retrograde cholangiopancreatography (DB-ERCP) differs among countries and/or facilities, and there is no established method. This study aimed to evaluate the efficacy of non-anesthesiologist-administered propofol (NAAP) sedation using a target-controlled infusion (TCI) system during DB-ERCP.

**Methods:**

This retrospective study was conducted between May 2017 and December 2020 at an academic center. One hundred and fifty-six consecutive patients who underwent DB-ERCP were sedated by gastroenterologists using diazepam (*n* = 77) or propofol with a TCI system (*n* = 79), depending on the period. The primary endpoint was a comparison of poor sedation rates between the two groups. Poor sedation was defined as a condition requiring the use of other sedative agents or discontinuation of the procedure. Secondary endpoints were sedation-related adverse events and risk factors for poor sedation.

**Results:**

Poor sedation occurred significantly more often in the diazepam sedation group (diazepam sedation, *n* = 12 [16%] vs. propofol sedation, *n* = 1 [1%]; *P* = 0.001). Vigorous body movements (3 or 4) (diazepam sedation, *n* = 40 [52%] vs. propofol sedation, *n* = 28 [35%]; *P* = 0.038) and hypoxemia (< 85%) (diazepam sedation, *n* = 7 [9%] vs. propofol sedation, *n* = 1 [1%]; *P* = 0.027) occurred significantly more often in the diazepam sedation group. In the multivariate analysis, age < 70 years old (OR, 10.26; 95% CI, 1.57–66.98; *P* = 0.015), BMI ≥ 25 kg/m2 (OR, 11.96; 95% CI, 1.67–85.69; *P* = 0.014), and propofol sedation (OR, 0.06; 95% CI, 0.01–0.58; *P* = 0.015) were associated factors for poor sedation.

**Conclusions:**

NAAP sedation with the TCI system during DB-ERCP was safer and more effective than diazepam sedation.

## Introduction

The endoscopic approach for biliary/pancreatic disease with postoperative bowel reconstruction makes it difficult to reach the papilla or perform hepaticojejunostomy (HJ)/pancreatojejunostomy (PJ) with conventional endoscopes [1], and so far percutaneous or surgical treatment has been selected. However, balloon-assisted endoscopy (BAE), which was developed for the diagnosis and treatment of small bowel disease, has enabled an endoscopic approach to treat biliary/pancreatic disease in patients with postoperative bowel reconstruction [2].

The double balloon-endoscopic retrograde cholangiopancreatography (DB-ERCP) procedure includes the process of reaching the papilla or anastomosis of HJ/PJ; thus, it takes longer than the usual endoscopic retrograde cholangiopancreatography (ERCP). Consequently, uncontrolled body movements and/or sedation-related adverse events occur easily, and some patients have to interrupt or discontinue the procedure because of inadequate sedation management. More adequate sedation is required to complete DB-ERCP. The American Society for Gastrointestinal Endoscopy (ASGE) guidelines recommend that anesthesia-administered sedation be considered in all complex endoscopic procedures [3]. A previous study from the United States (US) reported that anesthesiology-administered sedation (monitored anesthesia care without an endotracheal tube [MAC-WET] and general endotracheal anesthesia [GEA]) and endoscopist-directed sedation (EDS) have been performed in approximately 70% and 30% cases, respectively [4]. However in some countries, including Japan, gastroenterologists perform intravenous anesthesia without intubation due to shortage of anesthesiologists. The sedation method used during DB-ERCP varies considerably between countries and institutions, and there is no established method.

Propofol is a short-acting sedative with a rapid recovery profile compared to that of other sedatives, which allows the patient to be sedated and awakened quickly [5]. These advantages have resulted in an increased use of propofol worldwide [6]. A target-controlled infusion (TCI) system automatically controls the dose of sedative drugs through a computer-assisted infusion algorithm for pharmacokinetics to calculate the effect-site concentration [7, 8]. In the clinical environment, propofol is mainly used by entering age, weight, and target blood concentration. The use of a TCI system for propofol administration allows rapid induction and safe maintenance of an appropriate level of sedation, making it ideal for complicated procedures [9].

It was also reported that a TCI system for administration of propofol provides safe and effective sedation during ERCP [10]. Moreover, previous studies reported that non-anesthesiologist-administered propofol (NAAP) sedation with a TCI system during ERCP may be acceptable in elderly patients with a lower dose of propofol than that used in younger patients [7]. European guidelines state that NAAP can be safely applied in endoscopic procedures [11]; however there is no report about NAAP sedation during DB-ERCP. If DB-ERCP can be safely performed using NAAP with a TCI system, it may reduce the burden of general anesthesia on anesthesiologists and patients. Therefore, this study aimed to evaluate the efficacy of NAAP sedation using a TCI system during DB-ERCP.

## Materials and methods

### Patients

**Fig. 1:**
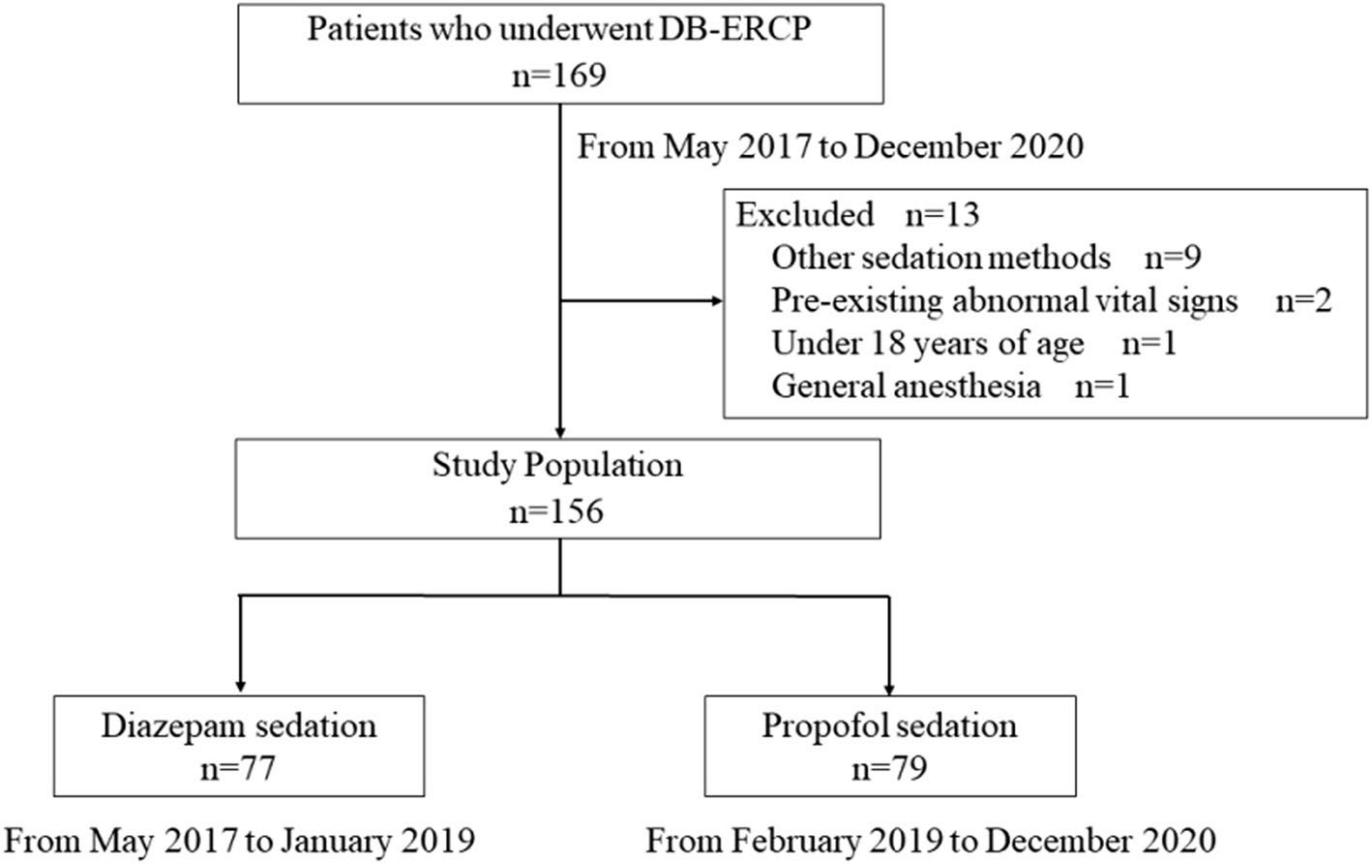
Diagram of the study design. *DB-ERCP*, double balloon-endoscopic retrograde cholangiopancreatography

One hundred and sixty-nine consecutive patients who underwent DB-ERCP at our institution between May 2017 and December 2020 were included in this retrospective study (Fig. 1). The inclusion criteria were as follows: (1) patients with altered anatomy (Child, including Pylorus-Preserving Pancreaticoduodenectomy-IIA and Subtotal Stomach-Preserving Pancreaticoduodenectomy-IIA, Roux-en-Y, or Billroth-II) and (2) patients who required detailed examination or treatment of the bile duct or pancreatic duct. Only the first procedure was included among patients who underwent multiple procedures during the study period. The exclusion criteria were: (1) patients under 18 years of age; (2) other sedation methods; (3) general anesthesia; (4) pre-existing hypotension (systolic blood pressure < 90 mmHg), bradycardia (heart rate < 50 /minute), hypoxemia (SaO2 < 90%), or the need for oxygen supplementation before the start of the sedation; (5) American Society of Anesthesiologists (ASA) class IV or higher. During the study period, 156 patients who underwent DB-ERCP met the inclusion criteria. At our institution, diazepam was used for sedation between May 2017 and January 2019 and propofol was used between February 2019 and December 2020. Among them, 77 patients who received diazepam sedation and 79 who received propofol sedation were analyzed (Fig. 1).

### Sedation protocol and monitoring

During the procedure, all patients were continuously monitored for heart rate, oxygen saturation, and electrocardiographic changes using a bedside monitor (BSM-2301; Nihon Kohden Wellness Corporation, Tokyo, Japan). The blood pressure was automatically assessed every 5 min. All patients received supplemental oxygen (2 L/min) via a nasal cannula during sedation and were maintained in the prone position. All procedures were performed using either of two types of short double-balloon endoscopes, EI-530B or EI-580BT (Fujifilm, Tokyo, Japan), with CO2 insufflation.

All medication and management procedures were performed by a gastroenterologist who did not directly participate in the procedures. The anesthesiologist was on standby in the event of an emergency.

- 1) Diazepam sedation

**Fig. 2:**
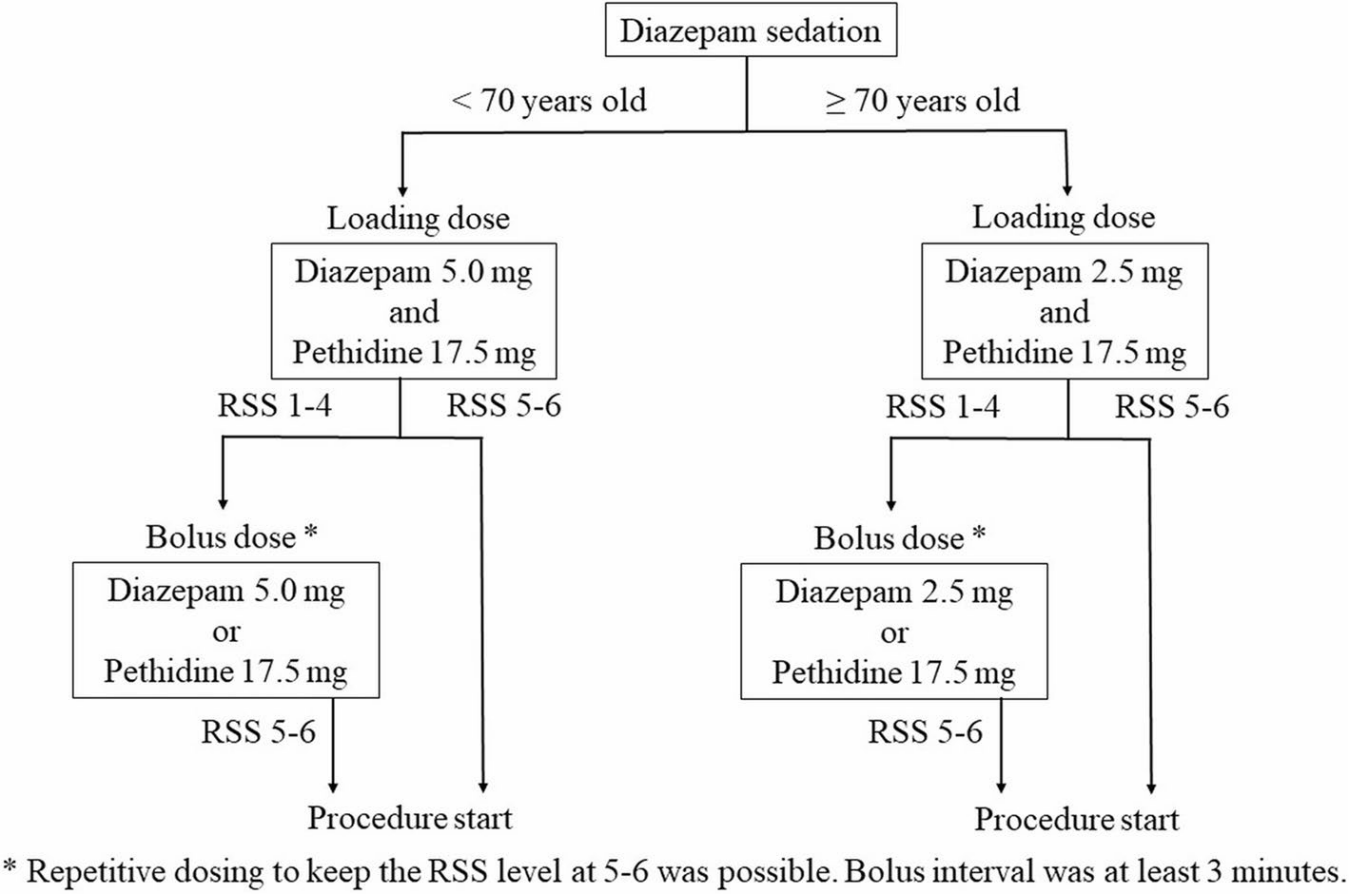
Flowchart of the protocol for diazepam sedation. *RSS*, Ramsay sedation scale

Basically, the loading dose or repeated doses of diazepam (Teva Takeda, Nagoya, Japan) were 5.0 mg for the non-elderly patients (< 70 years old) or 2.5 mg for the elderly patients (≥ 70 years old). After an intravenous loading dose of 2.5–5.0 mg diazepam and 17.5 mg pethidine (Takeda, Tokyo, Japan) had been injected, repeated doses of 2.5–5.0 mg diazepam or 17.5 mg pethidine were given intravenously targeting levels 5–6 of the Ramsay sedation scale (RSS, Table 1), which is equivalent to deep sedation. When body movements were frequent, bolus dose of 2.5–5.0 mg diazepam or 17.5 mg of pethidine was injected. Maximum doses of diazepam and pethidine were 20.0 and 140.0 mg, respectively (Fig. 2).

- 2) Propofol sedation

**Table 1:** Ramsay sedation scale

Score	Response
1	Anxious, agitated, restless
2	Cooperative, oriented, tranquil
3	Responsive to commands only
4	Brisk response to light glabellar tap or loud auditory stimulus
5	Sluggish response to light glabellar tap or loud auditory stimulus
6	No response to light glabellar tap or loud auditory stimulus

**Fig. 3:**
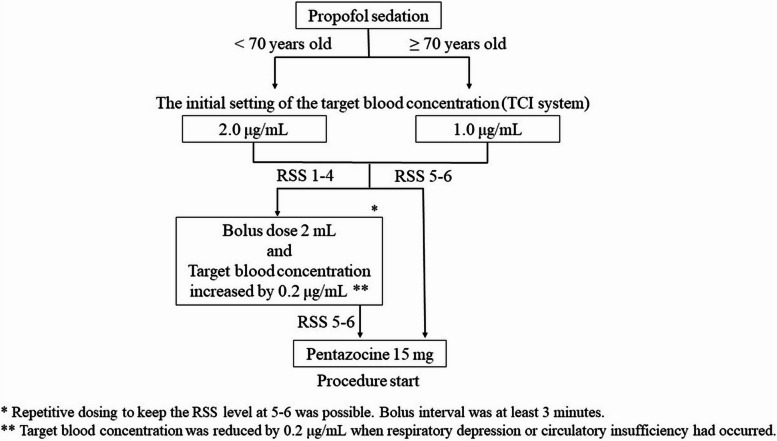
Flowchart of the protocol for propofol sedation. *TCI*, target-controlled infusion; *RSS*, Ramsay sedation scale

Propofol was administered intravenously using a Diprifusor system (TE-371; Terumo, Tokyo, Japan), which is a TCI system. The initial setting of the target blood concentration of propofol (1% Diprivan injection-kit; AstraZeneca, Osaka, Japan) was set at 2.0 μg/mL for the non-elderly patients (< 70 years old). The initial setting for elderly patients (≥ 70 years) was 1.0 μg/mL. These doses were chosen based on previous studies [12–15]. As an analgesic, a dose of 15 mg pentazocine (Maruishi, Osaka, Japan) was intravenously injected immediately before scope insertion. An RSS (Table 1) of 5–6 was considered the appropriate sedation level. When the RSS was 1–4, or body movements were frequent, a bolus dose of propofol (2 mL) was injected, and continuous infusion was increased by 0.2 μg/mL (Fig. 3). The dose of the propofol continuous infusion was reduced by 0.2 μg/mL when respiratory depression or circulatory insufficiency had occurred.

In both groups, when the sedation target level was less than RSS 4, we added a bolus dose of the same sedative agent that was initially used. The bolus interval was at least 3 min. If the target sedation level did not reach RSS 5–6, other sedative agents were added. The procedure was continued if the RSS level was maintained at 5–6. If not, the procedure was discontinued. The procedure was also discontinued if severe respiratory depression or circulatory insufficiency occurred.

### Outcomes

The primary endpoint was poor sedation, which we compared between the two groups. Poor sedation was defined as a condition requiring the use of other sedative agents to maintain the target sedation level (RSS 5–6) or the discontinuation of the procedure. The secondary endpoints were a comparison of the rate of sedation-related adverse events and an analysis of the risk factors for poor sedation. Sedation-related adverse events were defined as bradycardia (heart rate < 50/minute), hypotension (blood pressure < 90/50 mmHg or < 20%), or hypoxemia (SpO2 < 85%) [16]. We adopted the body movement score reported by Oshima et al. [17] Body movement scores of 4 to 5 were judged to indicate body movement (Table 2). We also compared the procedure time, infusion drug doses, procedure-related success rates, and post-ERCP pancreatitis rates. Procedure-related success was defined as reaching the HJ or PJ and completing a procedure. Post-ERCP pancreatitis was evaluated using the ASGE guideline [16].

**Table 2:** Score of body movement

Score	Response
1	No movement
2	Occasional, slight movement
3	Frequent, slight movement
4	Vigorous movement limited to extremities
5	Vigorous movement, including torso and head

### Statistical analysis

Continuous variables are expressed as mean ± standard deviation (SD). The Chi-square test was used to analyze categorical variables. The Mann–Whitney U test was used to compare continuous variables. Multivariate analysis was performed using logistic regression to identify significant risk factors for poor sedation. For variable selection, significant variables in the univariate analysis (*P* < 0.05) were selected for inclusion in the multivariate model. The significance level was set at *P* < 0.05.

## Results

### Patient characteristics

The mean age of the patients was 67.8 ± 13.5 years. There were no significant differences between the two groups in terms of age, sex, body mass index (BMI), current or ex-smoker status, alcohol abuse, regular narcotic/sedative use, comorbidities, ASA class, bowel reconstruction methods, or indications. There were no significant differences in baseline vital signs between the two groups (Table 3).

**Table 3:** Characteristics of study patients

	All patients (*n* = 156)	Diazepam sedation (*n* = 77)	Propofol sedation (*n* = 79)	*P* value
Age, mean ± SD, years	67.8 ± 13.5	68.7 ± 13.5	66.9 ± 13.6	0.256
Sex, male/female	94/62	45/32	49/30	0.648
BMI, mean ± SD, kg/m2	20.6 ± 3.4	21.1 ± 4.0	20.1 ± 2.8	0.162
Current or ex-smoker, n (%)	76 (49)	33 (43)	43 (54)	0.148
Alcohol abuse, n (%)	36 (23)	22 (29)	14 (18)	0.108
Regular narcotic/sedative use, n (%)	33 (21)	12 (16)	21 (27)	0.093
Co-morbidities, n (%)				
Heart disease	18 (12)	10 (13)	8 (10)	0.576
Lung disease	14 (9)	6 (8)	8 (10)	0.610
Renal disease	7 (4)	4 (5)	3 (4)	0.673
Liver disease	15 (10)	5 (6)	10 (13)	0.192
ASA class, n (%)				0.235
1	40 (26)	24 (31)	16 (20)	
2	95 (61)	42 (55)	53 (67)	
3	21 (13)	11 (14)	10 (13)	
Bowel reconstruction methods, n (%)				0.309
Child	68 (53)	39 (51)	40 (51)	
Roux-en-Y	53 (41)	32 (42)	37 (47)	
Billroth-II	8 (6)	6 (8)	2 (3)	
Indications, n (%)				0.717
Biliary stenosis	52 (33)	23 (30)	29 (37)	
Cholangitis	37 (24)	18 (23)	19 (24)	
Biliary stones	36 (23)	19 (25)	17 (22)	
Obstructive jaundice	4 (3)	2 (3)	2 (3)	
Biliary leaks	4 (3)	1 (1)	3 (4)	
Others	23 (15)	14 (18)	9 (11)	
Baseline heart rate, mean ± SD, beats/minute	71.5 ± 12.4	69.7 ± 11.0	73.3 ± 13.5	0.086
Baseline SBP, mean ± SD, mmHg	122.1 ± 16.8	120.7 ± 19.5	123.5 ± 13.6	0.083
Baseline oxygen saturation, mean ± SD, %	97.5 ± 1.3	97.3 ± 1.4	97.6 ± 1.2	0.110

*SD* standard deviation, *BMI* body mass index, *ASA* American Society of Anesthesiologists, *SBP* systolic blood pressure

### Infusion drug doses and sedation-related adverse events

Procedure time and procedure-related success rate did not differ between the two groups (diazepam sedation: 68.7 ± 40.1 min versus propofol sedation: 59.0 ± 26.3 min; *P* = 0.159, diazepam sedation, 63 [82%] versus propofol sedation, 65 [82%]; *P* = 0.940). None of the patients developed post-ERCP pancreatitis. Poor sedation occurred significantly more often in the diazepam sedation group (diazepam sedation, *n* = 12 [16%] vs. propofol sedation, *n* = 1 [1%]; *P* = 0.001). All 12 patients in the diazepam group with poor sedation completed the procedure with additional propofol. One patient in the propofol sedation group with poor sedation discontinued because of hypoxemia. In terms of sedation-related adverse events, vigorous body movements (4 or 5) (diazepam sedation, *n* = 40 [52%] vs. propofol sedation, *n* = 28 [35%]; *P* = 0.038) and hypoxemia (< 85%) (diazepam sedation, *n* = 7 [9%] vs. propofol sedation, *n* = 1 [1%]; *P* = 0.027) occurred significantly more often in the diazepam sedation group. There were no significant differences between the two groups in terms of bradycardia (< 50/minute) (diazepam sedation: *n* = 7 [9%] vs. propofol sedation: *n *= 4 [5%]; *P* = 0.326) and hypotension (< 90/50 mmHg or < 20%) (diazepam sedation: *n* = 13 [17%] vs. *n* = 21 [27%]; propofol sedation: *n* = 19 [27%]; *P* = 0.142) (Table 4).

**Table 4:** Infusion drug doses and sedation-related adverse events

	Diazepam sedation (*n* = 77)	Propofol sedation (*n* = 79)	*P* value
Procedure time, mean ± SD, minutes	68.7 ± 40.1	59.0 ± 26.3	0.159
Total infusion dose of diazepam, mean ± SD, mg	9.7 ± 4.6		
Total infusion dose of pethidine, mean ± SD, mg	93.6 ± 33.2		
Total infusion dose of propofol, mean ± SD, mg		317.0 ± 145.9	
Total infusion dose of pentazocine, mean ± SD, mg		15.0 ± 0	
Procedure-related success, n (%)	63 (82)	65 (82)	0.940
Post-ERCP pancreatitis, n (%)	0 (0)	0 (0)	-
Poor sedation, n (%)	12 (16)	1 (1)	0.001
Required other sedative agents/discontinued the procedure	12/0	0/1	
Vigorous body movement (4 or 5), n (%)	40 (52)	28 (35)	0.038
Bradycardia (< 50/minute), n (%)	7 (9)	4 (5)	0.326
Hypotension (< 90/50 mmHg or down 20%), n (%)	13 (17)	21 (27)	0.142
Hypoxemia (< 85%), n (%)	7 (9)	1 (1)	0.027

*SD* standard deviation, *ERCP* endoscopic retrograde cholangiopancreatography

### Risk factors for poor sedation

Table 5 shows the univariate and multivariate analyses of risk factors for poor sedation. In the univariate analysis, age < 70 years old (odds ratio [OR], 4.48; 95% confidence interval [CI], 1.18–16.98; *P* = 0.027), BMI ≥ 25 kg/m2 (OR, 16.65; 95% CI, 4.41–62.90; *P* < 0.0001), Roux-en-Y anastomosis (OR, 8.06; 95% CI, 1.72–37.72; *P* = 0.008), procedure time ≥ 60 min (OR, 13.61; 95% CI, 1.72–107.47; *P* = 0.013), and propofol sedation (OR, 0.07; 95% CI, 0.01–0.55; *P* = 0.011) were significant factors for poor sedation (*P* < 0.05). In the multivariate analysis, age < 70 years old (OR, 10.26; 95% CI, 1.57–66.98; *P* = 0.015), BMI ≥ 25 kg/m^2^ (OR, 11.96; 95% CI, 1.67–85.69; *P* = 0.014), and propofol sedation (OR, 0.06; 95% CI, 0.01–0.58; *P* = 0.015) were associated factors for poor sedation.

**Table 5:** Risk factors for poor sedation

	n	Number of patients with poor sedation	Univariate analysis			Multivariate analysis		
			OR (95% CI)		*P* value	OR (95% CI)		*P* value
Age < 70 years old	71	10	4.48	(1.18–16.98)	0.027	10.26	(1.57–66.98)	0.015
Sex, male	94	7	0.75	(0.24–2.35)	0.623			
BMI ≥ 25 kg/m2	13	6	16.65	(4.41–62.90)	< 0.0001	11.96	(1.67–85.69)	0.014
Current or ex-smoker	76	6	0.89	(0.29–2.79)	0.847			
Alcohol abuse	36	5	2.26	(0.69–7.39)	0.178			
Regular narcotic/sedative use	33	1	0.29	(0.04–2.31)	0.242			
Heart disease	18	1	0.62	(0.08–5.05)	0.653			
Lung disease	14	1	0.83	(0.10–6.93)	0.866			
Renal or liver disease	21	2	1.19	(0.24–5.77)	0.832			
ASA class 3	21	3	2.08	(0.52–8.29)	0.298			
Roux-en-Y anastomosis	69	11	8.06	(1.72–37.72)	0.008	3.80	(0.65–22.39)	0.140
Procedure time ≥ 60 min	79	12	13.61	(1.72–107.47)	0.013	4.72	(0.49–45.08)	0.178
Cholangitis	42	2	0.46	(0.10–2.19)	0.331			
Propofol sedation	79	1	0.07	(0.01–0.55)	0.011	0.06	(0.01–0.58)	0.015

*OR* odds ratio, *CI* confidence interval, *BMI* body mass index, *ASA* American Society of Anesthesiologists

## Discussion

To the best of our knowledge, this is the first study to report the efficacy of propofol sedation using a TCI system during DB-ERCP. The incidences of poor sedation, vigorous body movement, and hypoxemia in patients under propofol sedation were significantly lower than those in patients under diazepam sedation. Age < 70 years, BMI ≥ 25 kg/m^2^, and propofol sedation were associated with poor sedation in multivariate analysis. Using a TCI system, NAAP sedation was effective and safe even in DB-ERCP procedures.

The advantages of propofol are short-acting and early awakening pharmacokinetic characteristics and adjustable depth of sedation [18–22]. In previous reports of ERCP, propofol provided the same or superior sedation quality as midazolam in terms of better patient cooperation and shorter recovery time [10, 23–27]. The disadvantage of propofol is that once cardiorespiratory inhibition has occurred, it is necessary to provide cardiorespiratory support until propofol is metabolized because of no available antagonists [13]. Benzodiazepines, such as diazepam, midazolam, alprazolam, and bromazepam are among the most commonly used drugs [21]. One of the major advantages of benzodiazepines is that the recovery time can be shortened by using the benzodiazepine antagonist flumazenil [28]. Moderate sedation with benzodiazepines and opioids is still considered the standard method of sedation. However, propofol usage is increasing in many countries because both the endoscopists’ and patients’ satisfaction is higher than with conventional sedation [29].

Several studies have demonstrated the usefulness of propofol sedation using a TCI system during ERCP. Ogawa et al. reported that safe sedation can be achieved even in elderly patients by reducing the propofol dose using a TCI system [7].

Mazanikov et al. reported that both TCI and patient-controlled sedation (PCS) are acceptable methods of propofol administration during ERCP with high success rates and similar adverse event profiles [30]. European guidelines recommended administering propofol through intermittent bolus infusion or perfusor systems including a TCI system during NAAP sedation [11]. However, there have been no reports on the usefulness of a TCI system during DB-ERCP.

The study found that poor sedation was less frequent in the propofol group than in the diazepam group (*P* = 0.001). Poor sedation occurred in 12 (16%) patients with diazepam sedation, and all of them were able to complete the procedure with additional propofol. Poor sedation with propofol occurred in only one case (1%), and the procedure was discontinued due to hypoxemia. The patient required temporary ventilatory support after discontinuation of the procedure; however, his respiratory status improved rapidly.

The risk factors for poor sedation were age < 70 years, BMI ≥ 25 kg/m2, and diazepam sedation. The median age [interquartile range (IQR)] of the 13 patients with poor sedation was 57 (32–68) years. Non-elderly people generally tend to be less susceptible to sedation [18]. This is because drug metabolism declines with increasing age. With this in mind, we adopted a sedation protocol of increasing the dosage of sedative agents (diazepam or propofol) in the non-elderly group (age < 70 years). The dosage of propofol was significantly higher in the non-elderly group (non-elderly group: 381 ± 154 mg versus elderly group: 251 ± 102 mg; *P* < 0.0001). In contrast, the dosage of diazepam did not differ between the two groups (non-elderly group: 10.5 ± 4.9 mg versus elderly group: 9.2 ± 4.3 mg; *P* = 0.250).

Because the effect-site concentration of propofol could be monitored using the TCI system, an appropriate sedation dose could be administered to patients under propofol sedation. The under-administration of diazepam in the non-elderly group might have contributed to poor sedation. In obese patients, fat-soluble drugs such as diazepam and propofol are likely to migrate to the adipose tissue. Drug clearance increases owing to increased hepatic blood flow and cardiac output associated with obesity. These reasons may have led to a lower dosage of diazepam. On the other hand, it was expected that a proper dosage of propofol be maintained by body weight correction with a TCI system. Body movements (4 or 5) occurred significantly less often in the propofol sedation group (diazepam sedation, *n* = 40 [52%] versus propofol sedation, *n* = 28 [35%]; *P* = 0.038). The reason for the decrease in poor sedation with propofol is thought to be that the continuous intravenous infusion of propofol maintained an appropriate depth of sedation and reduced body movement. As a result, the propofol sedation using a TCI system became a protective factor against poor sedation in the multivariate analysis.

In terms of sedation-related adverse events, hypoxemia (< 85%) occurred significantly more often in the diazepam sedation group (diazepam sedation, *n* = 7 [9%] vs. propofol sedation, *n* = 1 [1%]; *P* = 0.027). The reason for this was thought to be the difficulty in appropriately adjusting the bolus dose of diazepam. Patients who develop hypoxemia were only managed by increasing the oxygen flow rate. There were no differences between the two groups in the rates of bradycardia (< 50/minute) (*P* = 0.326) or hypotension (< 90/50 mmHg or < 20%) (*P* = 0.142). All patients who developed bradycardia and hypotension were managed by increasing the infusion rate or decreasing the blood propofol concentration.

BAE includes double-balloon endoscopy (DBE) and single-balloon endoscopy (SBE). DBE tends to achieve a deeper insertion depth by better anchoring the intestine using a balloon on the scope tip. In contrast, SBE has a shorter preparation time because it does not require mounting a balloon on the tip of the scope. In some cases, the SBE can be inserted by a single endoscopist. A multicenter retrospective study showed that the success rate of endoscopic retrograde cholangiopancreatography (ERCP) in patients who underwent Roux-en-Y was found to be similar for both DBE and SBE [31]. In our hospital, only DBE has been used in patients with altered anatomy. Thus, we could not compare the two scopes in this study.

Our study has several limitations. First, it was a retrospective study. However, the bias was minimized by accumulating consecutive cases using the same protocol for each period. Second, analgesics were not combined with pethidine for diazepam sedation or pentazocine for propofol sedation. Additional infusions of pethidine were administered only under diazepam sedation. Therefore, differences in analgesics may be responsible for poor sedation or adverse events.

## Conclusion

In conclusion, NAAP sedation with a TCI system during DB-ERCP is a safer and more useful method with fewer cases of poor sedation than diazepam sedation. Further large-scale studies with prospective controlled designs are required to standardize propofol sedation.

## Data Availability

The datasets used and/or analyzed in the current study are available from the corresponding author upon reasonable request.

## Abbreviations

HJ: Hepaticojejunostomy
PJ: Pancreatojejunostomy
BAE: Balloon-assisted endoscopy
DB-ERCP: Double balloon-endoscopic retrograde cholangiopancreatography
ERCP: Endoscopic retrograde cholangiopancreatography
ASGE: American Society for Gastrointestinal Endoscopy
US: United States
MAC-WET: Monitored anesthesia care without an endotracheal tube
GEA: General endotracheal anesthesia
EDS: Endoscopist-directed sedation
TCI: Target-controlled infusion
NAAP: Non-anesthesiologist-administered propofol
ASA: American Society of Anesthesiologists
RSS: Ramsay sedation scale
SD: Standard deviation
BMI: Body mass index
OR: Odds ratio
CI: Confidence interval
IQR: Interquartile range
PCS: Patient-controlled sedation
DBE: Double-balloon endoscopy
SBE: Single-balloon endoscopy
